# Development a nomogram to predict fertilisation rate of infertile males with borderline semen by using semen parameters combined with AMH and INHB

**DOI:** 10.1111/and.14182

**Published:** 2021-07-16

**Authors:** Jialing Li, Ting Hu, Yanfei Wang, Yunxing Fu, Feimiao Wang, Rong Hu

**Affiliations:** ^1^ Ningxia Medical University Yinchuan China; ^2^ Key Laboratory of Fertility Preservation and Maintenance of Ministry of Education Ningxia Medical University Yinchuan China; ^3^ Gansu Provincial Maternity and Child‐care Hospital Lanzhou, Gansu China; ^4^ Reproductive Medicine Center General Hospital of Ningxia Medical University Yinchuan China

**Keywords:** AMH, fertilisation rate, INHB, nomogram

## Abstract

The sperm quality of some males is in a critical state, making it hard for clinicians to choose the suitable fertilisation methods. This study aimed to develop an intelligent nomogram for predicting fertilisation rate of infertile males with borderline semen. 160 males underwent in vitro fertilisation (IVF), 58 of whom received rescue ICSI (R‐ICSI) due to fertilisation failure (fertilisation rate of IVF ≤30%). A least absolute shrinkage and selection operator (LASSO) regression analysis identified sperm concentration, progressively motile spermatozoa (PMS), seminal plasma anti‐Müllerian hormone (spAMH), seminal plasma inhibin (spINHB), serum AMH (serAMH) and serum INHB (serINHB) as significant predictors. The nomogram was plotted by multivariable logistic regression. This nomogram‐illustrated model showed good discrimination, calibration and clinical value. The area under the receiver operating characteristic curve (AUC) of the nomogram was 0.762 (*p* < .001). Calibration curve and Hosmer–Lemeshow test (*p* = .5261) showed good consistency between the predictions of the nomogram and the actual observations, and decision curve analysis showed that the nomogram was clinically useful. This nomogram may be useful in predicting fertilisation rate, mainly focused on new biomarkers, INHB and AMH. It could assist clinicians and laboratory technicians select appropriate fertilisation methods (IVF or ICSI) for male patients with borderline semen.

## 1 INTRODUCTION

The risk of total fertilisation failure (TFF) or near‐TFF is unavoidable during the conventional IVF treatment. The occurrence of TFF is still hard to predict, and incidence has been reported to range from 5% to as high as 15%–20% during the conventional IVF cycles (Combelles et al., 2010; Huang et al., 2014, 2015; Mahutte & Arici, 2003; Vitek et al., 2013), resulting no embryos being transferred and cycle cancellation, ultimately incurring a high emotional and financial toll on affected couples. Under such circumstance, rescue ICSI (R‐ICSI) has been used to overcome TFF or low fertilisation rate. Conventional IVF is performed 3 hr following oocyte retrieval, and cumulus–corona–oocyte complexes were inseminated by exposing them to spermatozoa for 3 hr. Then, laboratory operators check if oocytes extrude the second polar body. Those in which a second polar body is not extruded were subjected to early R‐ICSI after 6 hr of insemination (9 hr after oocyte retrieval) with fertilisation being checked on the next day (Chen & Kattera, 2003; Nagy et al., 2006), which is considered to be an effective approach to managing unfertilised oocytes. R‐ICSI is used as a routine remedy for in vitro fertilisation by a great many world's reproductive centres and has an ideal clinical outcome; however, early R‐ICSI involves mechanical operation of stripping granulosa cells from oocytes and delays several hours insemination compared with conventional ICSI (Chen et al., 2014). Although R‐ICSI does not affect the formation of the female–male pronucleus at this moment, some cytokines or mRNAs which maintain normal division of the embryo have been degraded or modified, thereby losing the original function and affecting the embryos’ quality and developmental potential (Sirard et al., 2006). In the present study, it was shown that compared with ICSI, high embryo quality rate, available embryos rate and blastocyst formation rate of the group of R‐ICSI were lower (Ping et al., 2017). Besides, polyspermia is more likely to occur in R‐ICSI. Even the rate of stillbirths and perinatal deaths are also higher for the R‐ICSI procedure relative to ICSI (Chen et al., 2014).

Semen analysis, which can evaluate semen quality, is a basic examination item for evaluating male fertility before clinical assisted reproductive technology (ART) treatment. It also has a certain reference value for the selection of assisted reproductive method and prediction of ART outcomes. However, single or comprehensive semen parameters still do not accurately predict the conception potential of ART (Bo et al., 2014; Wang & Swerdloff, 2014). In assisted reproduction programmes, decisions concerning the treatment technique (IVF or ICSI) are usually made after the evaluation of male fertility factors or taking into account the results of previous IVF attempts. Because ICSI has been recognised for its clinical effects, at present, the use of ICSI to treat male infertility has an increasingly common trend. However, in clinical practice, the indications of ICSI should be strictly controlled, and ICSI is supposed to be regarded as the last resort after the failure of conventional treatment. There is controversy over whether the critical semen quality needs to relax the ICSI indications. However, if such patients undergo conventional IVF, TFF may occur. Even if the semen of some patients can be remedied for R‐ICSI, it is difficult to save the fertilisation outcome. There are no widely accepted criteria, so the decision for males with borderline semen is often empirical, which may lead to complete fertilisation failure after IVF, or to the unnecessary use of ICSI and excessive treatment. Severe oligoasthenospermia is also easy to diagnose and choose ICSI to complete fertilisation through evaluating sperm parameters. In patients with mild and moderate oligoasthenospermia, which is called borderline semen, selecting whether IVF or ICSI will be conducted is essential.

Therefore, in the era background of precision medicine, it is necessary to find a method for accurately predicting the fertilisation ability of spermatozoa, and the selection of fertilisation methods is clinically required. If we can predict the fertilisation rate in advance, it is possible to avoid the adverse outcome caused by R‐ICSI after IVF and the excessive medical treatment caused by unnecessary ICSI. In our study, we choose some new efficient biomarkers. It is common knowledge that AMH and INHB belong to the transforming growth factor‐β (TGF‐β) superfamily, and all produced by the Sertoli cells (Barbotin et al., 2015; Josso, 2019) have a synergistic effect on the evaluation of male fertility.

Hence, the aim of this study was, therefore, to develop a nomogram using new biomarkers to predict the fertilisation rate of IVF to choose the appropriate fertilisation method for male patients with borderline semen.

## 2 PATIENTS AND METHODS

### 2.1 Subjects

The study population comprised 160 infertile couples, including 102 couples who only received IVF, and 58 couples who underwent R‐ICSI after the same cycles of IVF attributed to the low fertility rate of 30%. All participants were come from Reproductive Medicine Center of General Hospital of Ningxia Medical University from January 2017 to January 2019. Both husband and wife were ≤35 years of age, with a body mass index (BMI) between 19.0 and 26.0 kg/m^2^, and have complete medical history, normal chromosomes, no recent infection, chronic diseases disease and no adverse environmental exposure. The inclusion criterion of female was as follows: with two ovaries, normal level of sex hormone and 6–15 eggs were obtained in this cycle. The exclusion criterion of female was as follows: with polycystic ovarian syndrome (PCOS), endometriosis, a history of ovarian surgery and other abnormal ovarian function. The inclusion criterion of male as follows: different degrees of oligoasthenospermia, absence of cryptorchidism, varicocele, testicular trauma and azoospermia. This study was approved by the Reproductive Medicine Ethics Committee of General Hospital of Ningxia Medical University. All patients were informed of the purpose and methods of the experiment and signed the informed consent.

### 2.2 Assisted reproduction process

All female patients received intramuscular injection of GnRHa (Individualised treatment doses ranged from 1/3 to 1, Diphereline,) in the mid‐luteal phase of the previous cycle. Two weeks later, when the serum E2 level was<50 pmol/L, the endometrial thickness was <5 mm, serum FSH, LH were <5 IU/L, we use gonadotropin (Gn) to controlled ovarian hyperstimulation (COH). Ovarian stimulation achieved by administration of recombinant FSH (LIVON), initial dosage was 150–225 IU/day and FSH dosage in the light of the ovarian response assessed by transvaginal ultrasonography and serum E2 measurements. When three or more follicles ≥16 mm in diameter with a consistent rise in serum estradiol concentration, the patients were intramuscular injected human chorionic gonadotropin HCG (LIVON) 10 thousand units. 34–36 hr after HCG injection, oocyte aspiration was performed under vaginal ultra‐sound guidance. If sperm count of a male patient was <10 × 10^6^ spermatozoa/ml and the percentage of spermatozoa with forward motility <20%, ICSI was performed using standard procedures. When the above parameter is more than such values, conventional IVF was carried out, fertilisation process was evaluated after 4–6 hr of IVF by determining the number of polar bodies. When fertilisation rate is less than 30%, R‐ICSI would be performed.

### 2.3 Semen analysis

All subjects were asked to abstain from ejaculating for 2–7 days. Semen samples were obtained by masturbation on the day of ovum retrieval in a room adjacent to the semen laboratory. The sample was naturally liquefied in 37℃ thermostatic water bath, and semen volume and liquefaction time were recorded (complete liquefaction within 15 min, more than 60 min is considered abnormality). Sperm concentration and motility were evaluated using the CASA (computer‐assisted semen analysis operating system), in accordance with the WHO criteria (World Health Organization, 2010). Liquefied samples were taken 2 ml and centrifuged at 3,000 r/min for 10 min, and then, supernatant was collected 1ml, divided into 2 tubes, storied at −80℃. Smears of the samples for morphology observation were air dried, fixed in 95% ethanol and stained with the Papanicolaou method. Normal sperm morphology was determined according to the sperm morphological analysis of criteria of the 5th edition.

### 2.4 Hormone analysis

Blood samples were drawn from an antecubital vein between 8a.m. and 10a.m. and stored in separation gel tube. After standing for half an hour at room temperature (22℃), serum was centrifuged at 3000r/min for 10 min. Serum and seminal AMH and INHB concentration were measured by ELISA sandwich technique (All reagents were provided by Guangzhou Kangrun Biotechnology. Product batch number KR‐INHB‐001,2, KR‐AMH‐002,3). The reference value range of AMH is 0.06–18 ng/ml and INHB is 10–1,500 pg/ml. The intra‐assay coefficient of variation (CV) among the three batch kits was 15% (*n* = 10). Operate strictly in accordance with the product instructions.

### 2.5 Feature selection

A least absolute shrinkage and selection operator (LASSO) regression model with 10‐fold cross‐validation was used in order to minimise the risk of model overfitting. Optimisation of the *λ* parameter in the LASSO regression enables the coefficients of most features to be reduced to zero, with any features exhibiting nonzero coefficients being selected and retained for subsequent utilisation.

### 2.6 Nomogram construction

A nomogram was constructed and by generating risk scores for each patient based upon a linear combination of selected features and corresponding weighting coefficients from the LASSO analysis. These factors were used to generate a multivariable logistic regression model and a corresponding nomogram.

Nomogram accuracy was evaluated using calibration plots and assessments of discriminative ability. Area under curve of receiver operating characteristic (ROC) curve (AUC) values were computed in order to gauge nomogram discriminative capabilities. Model validation was conducted using the Hosmer–Lemeshow test. Furthermore, the clinical value of this nomogram was assessed through a decision curve analysis (DCA) approach wherein net benefits at different threshold probabilities were calculated.

### 2.7 Statistical analysis

All statistical tests were performed using R statistical software (version 3.3.2; R Foundation for Statistical Computing, Vienna, Austria). The ‘glmnet’ package was used for executing the LASSO algorithm. For the baseline characteristic analyses, all statistical analyses were performed using Statistical Program for Social Sciences (SPSS) version 17.0 for Windows (SPSS Inc, Chicago, IL). All statistical tests were two‐tailed, and *p* <.05 indicated a significant difference.

## 3 RESULTS

### 3.1 Comparison of clinical features and characteristics

The basic parameters are shown in Table 1. The average age of females in group of IVFS was 30.12 ± 2.98 years, and group of ICSI was 30.47 ± 3.54. No differences were found in the females mean age (*p* = .508). Similarly, there are no significant differences were found in the mean BMI of females (*p* = .184), mean age of males (*p* = .715) and BMI of males (*p* = .656). In group of IVFS, 9.74 ± 2.47 oocytes were retrieved in one cycle. Of those, about 7.50 ± 2.21 were at metaphase II. In group of R‐ICSI, 9.41 ± 2.26 oocytes were retrieved in one cycle and MII ovum number was 7.13 ± 2.15. The patients we included also did not show significant differences in these two indicators (*p* = .416; *p* = .316).

**TABLE 1:** The comparison of the basic parameters in group of IVF and R‐ICSI

Parameter	IVF (*x* ± *s*) (*n* = 102)	R‐ICSI(*x* ± *s*) (*n* = 58)	*p*
Females age (yr.)	30.12 ± 2.98	30.47 ± 3.54	0.508
Females BMI (kg/m^2^)	21.98 ± 2.17	22.42 ± 1.66	0.184
Males age (yr.)	32.86 ± 4.83	33.14 ± 4.10	0.715
Males BMI (kg/m^2^)	23.31 ± 1.67	23.19 ± 1.49	0.656
Infertile years	3.24 ± 2.17	3.38 ± 1.70	0.665
MII ovum number	7.50 ± 2.21	7.13 ± 2.15	0.316
Total oocytes retrieved	9.74 ± 2.47	9.41 ± 2.26	0.416

Note

Data were expressed as mean ±standard deviation (*SD*).

Abbreviations: BMI, body mass index.

### 3.2 Comparison of semen characteristics and hormone levels

As shown in Table 2, the fertilisation rate of IVF in R‐ICSI group was 0.24 ± 0.06, significantly lower the group of IVF group (0.62 ± 0.12). Seminal plasma AMH, INHB, serum INHB as well as total sperm count, sperm concentration and PMS are significant differences between groups. The other semen parameters, such as morphologically normal spermatozoa and sperm motility, were no significant difference between two groups.

**TABLE 2:** The comparison of Serum and seminal plasma AMH, INHB and semen parameters

Parameter	IVF (*x* ± *s*) (*n* = 102)	R‐ICSI (*x* ± *s*) (*n* = 58)	*p*
Fertilisation rate of IVF (%)	0.62 ± 0.12	0.24 ± 0.06	0.000^a^
Serum AMH (ng/ml)	10.49 ± 3.97	2.91 ± 2.56	0.000^a^
Seminal plasma AMH (ng/ ml)	2.43 ± 1.14	0.54 ± 0.41	0.000^a^
Serum INHB (pg/ml)	141.28 ± 33.72	111.75 ± 29.81	0.000^a^
Seminal plasma INHB (pg/ml)	60.05 ± 24.66	29.35 ± 5.94	0.000^a^
Total sperm count (×10^6^/ml)	137.15 ± 29.46	121.38 ± 40.04	0.010^a^
Sperm concentration (×10^6^/ml)	50.83 ± 10.87	45.24 ± 13.12	0.004^a^
Sperm motility (%)	53.07 ± 12.91	51.22 ± 8.63	0.281
PMS (%)	22.84 ± 4.40	16.55 ± 3.35	0.000^a^
MNS (%)	5.28 ± 1.08	5.17 ± 1.27	0.557

Note

Data were expressed as mean ±standard deviation (*SD*).

Abbreviations: AMH, anti‐Müllerian hormone; INHB: inhibin B; MNS: morphologically normal spermatozoa; PMS: progressively motile spermatozoa.

^a^ Significant difference between different groups.

### 3.3 Feature selection and parameters building

**FIGURE 1:**
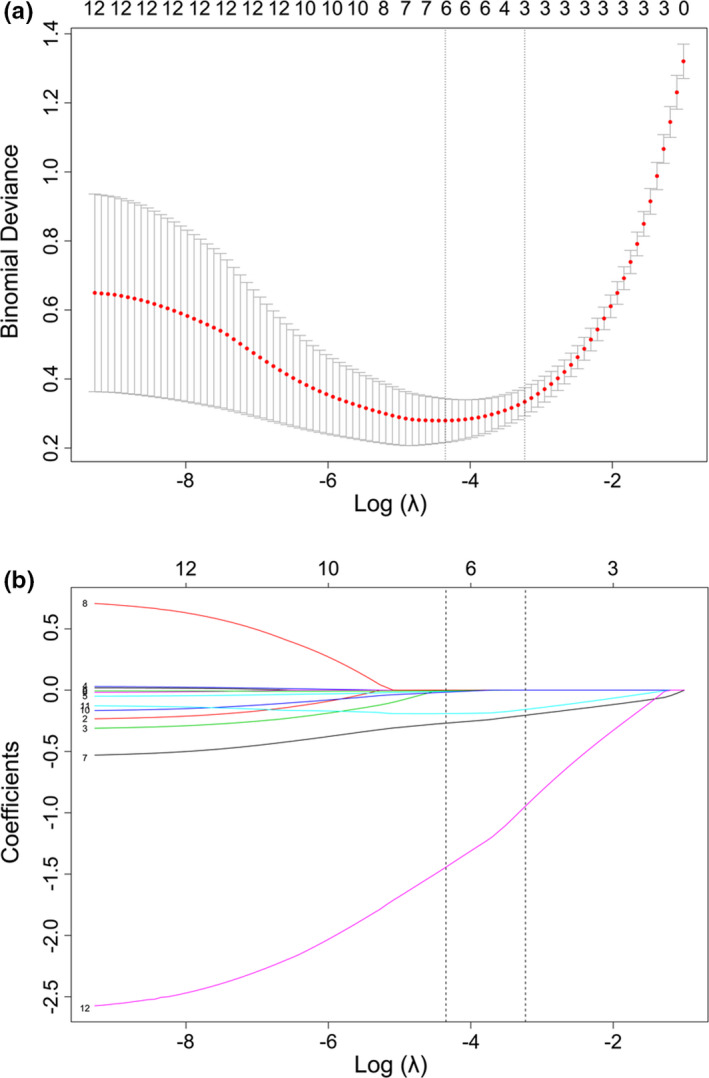
Radiomic feature selection using the least absolute shrinkage and selection operator (LASSO) regression method. (a) Selection of tuning parameters (l) in the LASSO model used 10‐fold cross‐validation via minimum criteria. The area under the curve was plotted versus log (λ). Dotted vertical lines were drawn at the optimal values using the minimum criteria and the 1‐standard error of the minimum criteria (the 1‐standard error criteria); (b) LASSO coefficient profiles of the radiomic features. A vertical line was plotted at the optimal λ value, which resulted in six features with nonzero coefficients

In order to identify key parameters associated with fertility rates in this patient cohort, we employed a LASSO regression approach that is well‐suited to analysing large number of clinical variables without the risk of overfitting. Risk scores were calculated for each patient based upon a linear combination of identified relevant factors and corresponding weighting coefficients. In total, we analysed 13 potential fertilisation rate‐related variables, of which 6 were ultimately suggested to be predictors of fertilisation rates by our LASSO regression model. Cross‐validated error plots corresponding to this regression model were generated (Figure 1a). Four variables were included in the most regularised model that exhibited cross‐validated error within a single standard error of the minimum, and path coefficients from this model with corresponding log‐transformed λ values were determined (Figure 1b).

### 3.4 Development of an individualised prediction model

**FIGURE 2:**
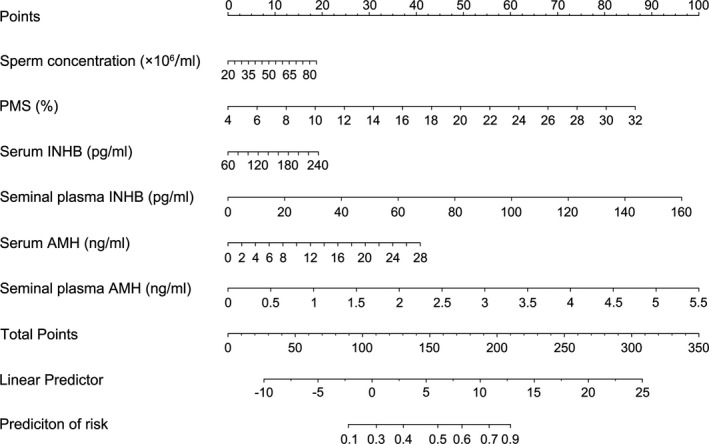
Nomogram for predicting fertilisation rate. Locate each variable on the corresponding axis and obtain a single score. Adding these single scores get the final total points. Draw a line straight up to find the fertilisation rate

The newly developed nomogram for predicting fertilisation rate using sperm concentration, PMS, spAMH, spINHB, serAMH and serINHB is demonstrated in Figure 2. Each point is first assigned by a vertical extension (to top points bar) of each parameter. The total point is obtained by adding scale for each variable. The total points projected on the bottom scale indicates the probability of fertilisation rate. The nomogram indicates that the probability of fertilisation rate increases with each variable. The equation of the nomogram is as follows:

The probability of fertilisation rate = *K*/(1 + *K*).

*K* = exp(16.38009 + 0.03598×V1+0.38436×V2+0.01328×V3+0.07493×V4+0.18145×V5+2.2623×V6).

### 3.5 Validation of the nomogram

**FIGURE 3:**
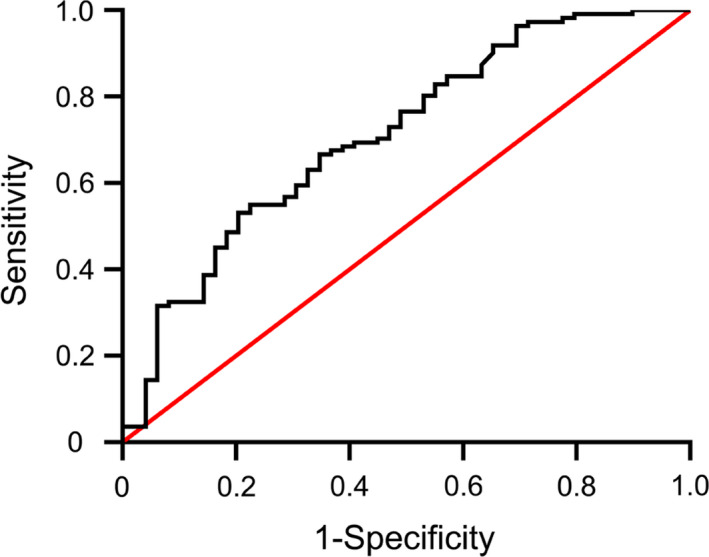
Receiver operating characteristic (ROC) curve of the nomogram. The area under the receiver operating characteristic curve (AUC) of the nomogram was 0.762 (95%CI: 62.4%‐80.0%, *p* <.001). The curved line is the ROC curve generated from the proposed multivariable prediction model, and the diagonal line is the reference line for random guessing

**FIGURE 4:**
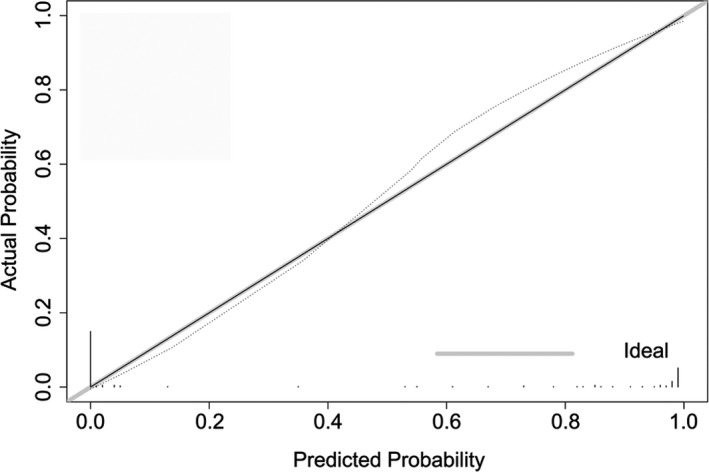
Calibration curves of nomogram. The y‐axis represents the actual fertilisation rate. The x‐axis represents the predicted fertilisation rate. The diagonal dotted line indicates perfect calibration based on an ideal model that would reflect the outcomes perfectly. The solid line indicates the nomogram's actual performance; a close alignment between the solid and dotted lines indicates better estimation of the actual outcomes

AUC analyses indicated that our model exhibited good discriminative potential, with an AUC of 0.762 (95%CI: 62.4%–80.0%, *p* <.001; Figure 3). Calibration curves for this nomogram exhibited good consistency between predicted and observed probability values (Figure 4), and no significant deviation was detected in Hosmer–Lemeshow goodness‐of‐fit tests (*p* =.5251).

**FIGURE 5:**
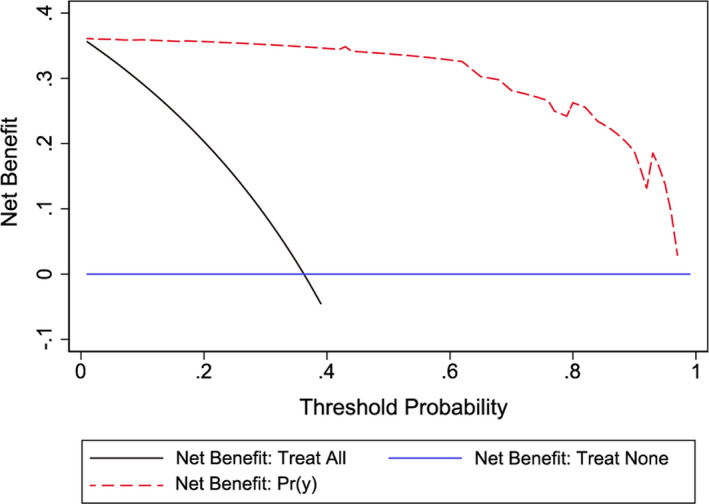
Decision curve analysis (DCA) for the predictive model. A horizontal blue line indicates that the fertilisation rate of all samples is 0, with a net benefit of zero. An oblique black line indicates the fertilisation rate of all patients is 1. The net benefit is a backslash with a negative slope

DCA is a novel method for evaluating alternative predictive strategies, which has advantages over the ROC curve. The DCA curve for the predictive nomogram is presented in Figure 5. The DCA curve showed obvious net benefits of the predictive nomogram.

## 4 DISCUSSION

Threshold values of sperm parameters for assisted procreation are based mainly on the World Health Organization standard and widely are used to discriminate between male fertility and subfertility (Hershlag et al., 2002; Pisarska et al., 1999; Verheyen et al., 1999). Unfortunately, no test can exclude the possibility of fertilisation failure. Severe oligoasthenospermia is easy to diagnose and choose ICSI to complete fertilisation through evaluating sperm parameters. But for the sperms of specific patients are between mild and moderate oligoasthenospermia, IVF is routinely used in the clinic. Once the fertilisation rate of IVF is low, R‐ICSI is adopted again. After these steps, the fertilisation outcomes may be poor, and fewer embryos will be available, especially for females who have obtained fewer eggs in this cycle. How to prevent fertilisation failure and maximise the use of eggs in an IVF cycle has always been the research priority in the field of assisted reproduction. R‐ICSI is the main measure for this situation. Compared with the conventional ICSI, the operation time of R‐ICSI after short‐term fertilisation often has a 5–6 hr lag, and the time of oocyte culture in vitro is significantly prolonged. Studies have shown that after 5–6 hr most oocytes have tended to age, missing the best period of fertilisation. At this time, oocytes can be fertilised by R‐ICSI, but it will affect functions of some cytokines or mRNA that maintain embryo cleavage, which may have a negative impact on embryo development potential (Grondahl et al., 2010; Leoni & Bebbere, 2006; Sirard et al., 2006). So, it is necessary to seek biomarkers for these patients with borderline semen, in order to predict sperm fertilisation ability and improve pregnancy outcomes. In this study, we established and validated an intelligent and practical nomogram as a new approach to select the appreciate fertilisation method for the male patients with borderline semen. To our knowledge, this is the first study to form a nomogram‐illustrated model to predict fertilisation rate. There were 13 candidate variables considered for the construction of the nomogram, which were reduced to 6 potential predictors, including sperm concentration, PMS, SerINHB, SpINHB, SerAMH and SpAMH, by the LASSO regression method. This method not only surpasses the method of choosing predictors on the basis of the strength of their univariable association with outcome (Harre et al., 1988), but also enables the panel of selected features to be combined into an integrated factor. The fifth edition of the routine semen analysis standard developed by WHO has been adopted by reproductive centres around the world to assess semen quality before assisted reproductive technology. In addition to these semen parameters, in recent years, serum AMH and INHB have been gradually used as parameters for assessing semen quality and male reproductive function. Originally, some scholars have found that serum AMH and INHB is associated with semen quality (Appasamy et al., 2007; Goulis et al., 2008; Grunewald et al., 2013). Similar to our study, serum AMH of patients with normal spermatozoa or mild asthenospermia (IVF group) appears to be extremely higher than serum AMH of patients with moderate oligozoospermia (R‐ICSI group). Besides, present study found that there were prominently differences in serum INHB between two groups, which is similar to Barbotin AL’s result (Barbotin et al., 2015).

As an important factor regulating male hypothalamic–pituitary–testicular gonadal axis, INHB specifically feedback regulates FSH, which is a sensitive indicator of spermatogenesis and a direct and effective endocrine marker for evaluating spermatogenesis. Its level reflects the number of spermatogenic cells (Mitchell et al., 2011). The study found that serum INHB has a high specificity in the identification of obstructive and nonobstructive azoospermia (Nagata et al., 2005) and positively correlated with forward motor spermatozoa and normal morphology sperm count (Barbotin et al., 2015), which can be used as a good biomarker for the diagnosis of male infertility (Manzoor et al., 2012). Additionally, AMH has both autocrine and paracrine properties through a direct effect via the AMH type II receptor and is therefore thought to be involved in spermatogenesis. AMH is closely related to the spermatogensis, but the specific relationship is not completely clear. Some studies have reported that there is a significant positive correlation between seminal plasma AMH and sperm concentration, total sperm count and sperm motility rate (Andersen et al., 2016), serum AMH level and sperm concentration, total sperm count, sperm activity rate and forward motor sperm rate are positively correlated (Long‐Ping et al., 2017). The latest research suggested seminal plasma AMH level was positively correlate with the semen characteristics as well as some other semen parameters such as intra‐acrosomal enzymes and anti‐spermatozoal antibodies. Among them, progressive sperm motility and sperm count has the strongest correlation (Andersen et al., 2016; Topolcan et al., 2016). In our research, we found that there were significant differences in seminal plasma AMH between two groups, and its variation tendency is the same as total sperm count, progressively motile spermatozoa as well as seminal plasma INHB. From here, we can draw a conclusion that serum and seminal plasma AMH, and INHB can also be indicators like sperm count and progressive sperm motility to determine the treatment protocols of ART for patients with different degree of oligoasthenospermia.

The key value of a nomogram is linked to its ability to accurately interpret a given individual's need for a particular form of medical care. However, the ability‐prediction performance, discrimination and calibration, alone cannot properly gauge the clinical outcomes of model miscalibration (Localio & Goodman, 2012; Karel et al., 2015; Van Calster & Vickers, 2015). As such, to justify the clinical usefulness, we included 160 male patients with oligoasthenospermia in the clinic and predict the fertilisation rate by the model. There were no statistical differences between the verification results and the clinical fertilisation rate, which means the model has certain credibility in choosing fertilisation methods and can be used in clinical application. But, the success of fertilisation also depends on the quality of oocytes. Thus, abnormalities of spermatozoa and eggs may cause low fertilisation rate and failure of fertilisation. The sample eggs that used to establish the model of this study were all from healthy young women of childbearing age, that is, the problem of fertilisation barrier caused by abnormal eggs was excluded. Therefore, this model may not be suitable for predicting the fertilisation rate and selecting fertilisation mode in vitro fertilisation for infertility female patients with advanced age, ovary dysfunction and polycystic ovary syndrome.

The accuracy and discrimination of the this nomogram have been verified, but in conventional IVF, defective sperm‐zona pellucida binding and penetration are the most common causes of failure of fertilisation in males with normal semen analysis and hormone levels (Liu et al., 2004). Mahutte and Arici (Mahutte & Arici, 2003) conducted a review of different screening tests. Their conclusion was that more sophisticated methods such as sperm‐zona binding ratios and zona pellucida‐induced acrosome reaction tests may improve the ability to predict fertilisation capacity. Liu et al. (Liu & Baker, 2003) have found 13% of these patients had a low fertilisation rate due to the abnormal sperm‐zonal band binding, while 21% had disordered zona pellucida‐induced acrosome reaction because of acrosome function defects. So, there are many other indicators that can be used as predictors of sperm quality and fertilisation ability, such as sperm DNA fragmentation index (DFI), phospholipase ζ (PLC ζ), sperm acrosin, fructose concentrations and Neutral α‐glucosidase in seminal plasma (Chaudhury et al., 2005; Giwercman et al., 2003; Khakpour et al., 2019). The activation of oocytes by spermatozoa is one of the key steps in fertilisation. Studies have shown IVF and ICSI failure is mostly caused by lack of oocyte activating factors in spermatozoa, which cannot activate the oocytes normally (Yanagida et al., 2006; Yeste et al., 2016). As an important sperm factor that activates oocytes, PLC ζ is a key factor leading male infertility. Because repeated changes in intracellular calcium (Ca2+) concentration are the most effective activation signals for oocytes (Saleh et al., 2020), besides, spermatozoa from men with proven oocyte activation capacity presented a significantly higher proportion of spermatozoa exhibiting PLC‐ζ immunofluorescence compared with infertile spermatozoa from men resulted in recurrent ICSI failure (oocyte activation deficient(Kashir et al., 2013). Studies have also shown that the expression level of PLC ζ mRNA and protein in male patients with round head spermatozoa is significantly lower than that of normal males (Chanseldebordeaux et al., 2015). In patients with azoospermia, PLC ζ is low expression or absence. The researchers found that measurement of DFI provides a simple, informative and reliable measure of sperm quality and can accurately predict male mouse fertility (Li & Lloyd, 2020), and sperm DFI was negatively correlated with sperm density, motility and normal morphological rate in infertility patients (Varghese et al., 2009). Low levels of oxygen free radicals adversely affect sperm activation, which in turn increases the damage of sperm DNA (Agarwal et al., 2008). All of these studies have been suggested that sperm DIF can be use as one of the key indicators for evaluating fertilising capacity. In addition, for infertile patients, sperm acrosome enzyme is a crucial basis and identification method for judging sperm quality, combined with the conventional parameters of semen, the sperm acrosin activity can be better evaluated sperm function and provide a basis for diagnosis and treat of the disease (Cui et al., 2000; Langlois et al., 2005).

As the most basic indicators of male fertility, routine semen parameters have the disadvantages of high volatility, strong subjectivity and susceptibility to various factors. In clinical practice, the semen of some patients is normal but still fails to fertilise, or it is ineffective after corresponding treatment, suggesting that there are limitations in the formulation of treatment plan relying solely on routine semen parameters, and it is necessary to find other parameters that can reflect sperm quality, establish new laboratory techniques to assess male fertility and predict the outcome of ART. In recent years, research hotspots have focused on AMH INHB, DFI, acrosome reaction, mitochondrial membrane potential, sperm membranes, neutral α‐glucosidase and fructose concentrations, which may become a key reference circle for the diagnosis and treatment of male infertility. Based on these, we are planning to combine the medical testing centre and molecular biology laboratory to include other indicators and more patient samples to establish a more accurate nomogram model for predicting fertilisation rate to choose personalised fertilisation method for male patients with borderline semen.

## 5 CONCLUSION

We establish an intelligent and practical model incorporating sperm concentration, PMS, SerINHB, SpINHB, SerAMH and SpAMH that could be conveniently used to predict fertilisation rate and a useful tool to choose the optimal fertilisation method for infertile males with borderline semen.

## CONFLICT OF INTERESTS

None of the authors have any conflicts of interest to declare.

## Data Availability

The data that support the findings of this study are available from the corresponding author upon reasonable request.

## References

[and14182-bib-0001] Agarwal, A. , Makker, K. , & Sharma, R. (2008). Clinical relevance of oxidative stress in male factor infertility: An update. American Journal of Reproductive Immunology, 59(12), 2–11. 10.1111/j.1600-0897.2007.00559.x 18154591

[and14182-bib-0002] Andersen, J. M. , Herning, H. , Witczak, O. , & Haugen, T. B. (2016). Anti‐Müllerian hormone in seminal plasma and serum: Association with sperm count and sperm motility. Human Reproduction, 31(8), 1662–1667. 10.1093/humrep/dew121 27220981

[and14182-bib-0003] Appasamy, M. , Muttukrishna, S. , Pizzey, A. , Ozturk, O. , Groome, N. P. , Serhal, P. , & Jauniaux, E. (2007). Relationship between male reproductive hormones, sperm DNA damage and markers of oxidative stress in infertility. Reproductive Biomedicine Online, 14(2), 159–165. 10.1016/s1472-6483(10)60783-3 17298717

[and14182-bib-0004] Barbotin, A.‐L. , Ballot, C. , Sigala, J. , Ramdane, N. , Duhamel, A. , Marcelli, F. , Rigot, J.‐M. , Dewailly, D. , Pigny, P. , & Mitchell, V. (2015). The serum inhibin b concentration and reference ranges in normozoospermia. European Journal of Endocrinology, 172(6), 669–676. 10.1530/EJE-14-0932 25740852

[and14182-bib-0005] Chanseldebordeaux, L. , Dandieu, S. , Bechoua, S. , & Jimenez, C. (2015). Reproductive outcome in globozoospermic men: Update and prospects. Journal of Andrology, 3(6), 1022–1034. 10.1111/andr.12081 26445006

[and14182-bib-0006] Chaudhury, K. , Das, T. , Chakravarty, B. , & Bhattacharyya, A. K. (2005). Acrosin activity as a potential marker for sperm membrane characteristics in unexplained male infertility. Fertility and Sterility, 83(1), 104–109. 10.1016/j.fertnstert.2004.06.063 15652894

[and14182-bib-0007] Chen, C. , & Kattera, S. (2003). Rescue ICSI of oocytes that failed to extrude the second polar body 6 h post‐insemination in conventional IVF. Human Reproduction, 18(10), 2118–2121. 10.1093/humrep/deg325 14507831

[and14182-bib-0008] Chen, L. , Xu, Z. , Zhang, N. Y. , Wang, B. , Chen, H. , Wang, S. , & Sun, H. (2014). Neonatal outcome of early rescue ICSI and ICSI with ejaculated sperm. Journal of Assisted Reproduction and Genetics, 31(7), 823–828. 10.1007/s10815-014-0245-9 24824350 PMC4096880

[and14182-bib-0009] Combelles, C. M. H. , Morozumi, K. , Yanagimachi, R. , Zhu, L. , Fox, J. H. , & Racowsky, C. (2010). Diagnosing cellular defects in an unexplained case of total fertilization failure. Human Reproduction, 25(7), 1666–1671. 10.1093/humrep/deq064 20472911

[and14182-bib-0010] Cui, Y. , Zhao, R. , Wang, Q. , & Zhang, Z. (2000). Determination of sperm acrosin activity for evaluation of male fertility. Asian Journal of Andrology, 2(3), 229–232. CNKI:SUN:YZNK.0.2000‐03‐010 11225983

[and14182-bib-0011] Giwercman, A. , Richthoff, J. , Hjollund, H. , Bonde, J. P. , Jepson, K. , Frohm, B. , & Spano, M. (2003). Correlation between sperm motility and sperm chromatin structure assay parameters. Fertility and Sterility, 80(6), 1404–1412. 10.1016/s0015-0282(03)02212-x 14667876

[and14182-bib-0012] Goulis, D. G. , Iliadou, P. K. , Tsametis, C. , Gerou, S. , Tarlatzis, B. C. , Bontis, I. , & Papadimas, I. (2008). Serum anti‐Müllerian hormone levels differentiate control from subfertile men but not men with different causes of subfertility. Gynecological Endocrinology, 24(3), 158–160. 10.1080/09513590701672314 17926161

[and14182-bib-0013] Grondahl, M. L. , Andersen, C. Y. , Bogstad, J. , Nielsen, F. C. , Meinertz, H. , & Borup, R. (2010). Gene expression profiles of single human mature oocytes in relation to age. Human Reproduction, 25(4), 957–968. 10.1093/humrep/deq014 20147335

[and14182-bib-0014] Grunewald, S. , Glander, H. , Paasch, U. , & Kratzsch, J. (2013). Age‐dependent inhibin B concentration in relation to FSH and semen sample qualities: A study in 2448 men. Reproduction, 145(3), 237–244. 10.1530/REP-12-0415 23315688

[and14182-bib-0015] Harre, F. E. , Lee, K. L. , & Pollock, B. G. (1988). Regression models in clinical studies: determining relationships between predictors and response. Journal of the National Cancer Institute, 80(15), 1198–1202. 10.1093/jnci/80.15.1198 3047407

[and14182-bib-0016] Hershlag, A. , Paine, T. , Kvapil, G. , Feng, H. , & Napolitano, B. (2002). In vitro fertilization‐intracytoplasmic sperm injection split: An insemination method to prevent fertilization failure. Fertility & Sterility, 77(2), 229–232. 10.1016/s0015-0282(01)02978-8 11821076

[and14182-bib-0017] Huang, B. , Li, Z. , Zhu, L. , Hu, D. , Liu, Q. , Zhu, G. , & Zhang, H. (2014). Progesterone elevation on the day of HCG administration may affect rescue ICSI. Reproductive Biomedicine Online, 29(1), 88–93. 10.1016/j.rbmo.2014.03.015 24813756

[and14182-bib-0018] Huang, B. , Qian, K. , Li, Z. , Yue, J. , Yang, W. , Zhu, G. , & Zhang, H. (2015). Neonatal outcomes after early rescue intracytoplasmic sperm injection: An analysis of a 5‐year period. Fertility and Sterility, 103, 10.1016/j.fertnstert.2015.02.026 25813286

[and14182-bib-0019] Josso, N. (2019). WOMEN IN REPRODUCTIVE SCIENCE: Anti‐Müllerian hormone: A look back and ahead. Reproduction, 158(6), F81–f89. 10.1530/rep-18-0602 30844753

[and14182-bib-0020] Kashir, J. , Jones, C. , Mounce, G. , Ramadan, W. M. , Lemmon, B. , Heindryckx, B. , & Child, T. (2013). Variance in total levels of phospholipase C zeta (PLC‐ζ) in human sperm may limit the applicability of quantitative immunofluorescent analysis as a diagnostic indicator of oocyte activation capability. Fertility and Sterility, 99(1), 107–117. 10.1016/j.fertnstert.2012.09.001 23040527

[and14182-bib-0021] Khakpour, S. , Sadeghi, E. , Tavalaee, M. , Bahadorani, M. , & Nasresfahani, M. H. (2019). Zeta method: A noninvasive method based on membrane charge for selecting spermatozoa expressing high level of phospholipaseCζ. Andrologia, 51(5), 10.1111/and.13249 30873668

[and14182-bib-0022] Langlois, M. , Oorlynck, L. , Vandekerckhove, F. , Criel, A. , Bernard, D. , & Blaton, V. (2005). Discrepancy between sperm acrosin activity and sperm morphology: Significance for fertilization in vitro. Clinica Chimica Acta, 351(1), 121–129. 10.1016/j.cccn.2004.08.001 15563880

[and14182-bib-0023] Leoni, G. G. , Bebbere, D. , Succu, S. , Berlinguer, F. , Mossa, F. , Galioto, M. , Bogliolo, L. , Ledda, S. , & Naitana, S. (2006). Relations between relative mRNA abundance and developmental competence of ovine oocytes. Molecular Reproduction & Development, 74(2), 249–257. 10.1002/mrd.20442 16941675

[and14182-bib-0024] Li, B. O. , Ma, Y. , Huang, J. , Xiao, X. , Li, L. I. , Liu, C. , Shi, Y. , Wang, D. , & Wang, X. (2014). Probing the effect of human normal sperm morphology rate on cycle outcomes and assisted reproductive methods selection. PLoS One, 9(11), e113392‐. 10.1371/journal.pone.0113392 25411962 PMC4239063

[and14182-bib-0025] Li, M. , & Lloyd, K. C. K. (2020). DNA fragmentation index (DFI) as a measure of sperm quality and fertility in mice. Scientific Reports, 10(1), 3833. 10.1038/s41598-020-60876-9 32123279 PMC7052244

[and14182-bib-0026] Liu, D. Y. , & Baker, H. W. G. (2003). Disordered zona pellucida–induced acrosome reaction and failure of in vitro fertilization in patients with unexplained infertility. Fertility and Sterility, 79(1), 74–80. 10.1016/S0015-0282(02)04555-7 12524067

[and14182-bib-0027] Liu, D. Y. , Garrett, C. , & Baker, H. W. G. (2004). Clinical application of sperm‐oocyte interaction tests in in vitro fertilization–embryo transfer and intracytoplasmic sperm injection programs. Fertility and Sterility, 82(5), 1251–1263. 10.1016/j.fertnstert.2003.10.057 15533339

[and14182-bib-0028] Localio, A. R. , & Goodman, S. (2012). Beyond the usual prediction accuracy metrics: reporting results for clinical decision making. Annals of Internal Medicine, 157(4), 294–295. 10.7326/0003-4819-157-4-201208210-00014 22910942

[and14182-bib-0029] Long‐Ping, P. , Yong, S. , Cen‐Cen, W. , & Zou, Z.‐C. (2017). [Correlation of serum anti‐Müllerian hormone with semen parameters]. National Journal of Andrology, 23, 531–535. 29722946

[and14182-bib-0030] Mahutte, N. G. , Gonzalez, J. A. , Jones, E. , Taylor, H. S. , Duleba, A. J. , & Sakkas, D. (2003). Failed fertilization: Is it predictable? Current Opinion in Obstetrics & Gynecology, 15(3), 211–218. 10.1016/S0015-0282(02)03907-9 12858108

[and14182-bib-0031] Manzoor, S. M. , Sattar, A. , Hashim, R. , Khan, F. A. , Younas, M. , Ali, A. , & Ijaz, A. (2012). Serum inhibin B as a diagnostic marker of male infertility. Journal of Ayub Medical College Abbottabad, 24, 113–116. 24669628

[and14182-bib-0032] Mitchell, V. , Robin, G. , Boitrelle, F. , Massart, P. , Marchetti, C. , Marcelli, F. , & Rigot, J. M. (2011). Correlation between testicular sperm extraction outcomes and clinical, endocrine and testicular histology parameters in 120 azoospermic men with normal serum FSH levels. International Journal of Andrology, 34(4), 299–305. 10.1111/j.1365-2605.2010.01094.x 20695924

[and14182-bib-0033] Moons, K. G. M. , Altman, D. G. , Reitsma, J. B. , Ioannidis, J. P. A. , Macaskill, P. , Steyerberg, E. W. , Vickers, A. J. , Ransohoff, D. F. , & Collins, G. S. (2015). Transparent reporting of a multivariable prediction model for individual prognosis or diagnosis (TRIPOD): Explanation and elaboration. Annals of Internal Medicine, 162(1), W1. 10.7326/m14-0698 25560730

[and14182-bib-0034] Nagata, Y. , Fujita, K. , Banzai, J. , Kojima, Y. , Kasima, K. , Suzuki, M. , & Tanaka, K. (2005). Seminal plasma inhibin‐B level is a useful predictor of the success of conventional testicular sperm extraction in patients with non‐obstructive azoospermia. Journal of Obstetrics and Gynaecology Research, 31(5), 384–388. 10.1111/j.1447-0756.2005.00306.x 16176504

[and14182-bib-0035] Nagy, Z. P. , Rienzi, L. , Ubaldi, F. M. , Greco, E. , Massey, J. B. , & Kort, H. I. (2006). Effect of reduced oocyte aging on the outcome of rescue intracytoplasmic sperm injection. Fertility and Sterility, 85(4), 901–906. 10.1016/j.fertnstert.2005.09.029 16580372

[and14182-bib-0036] Ping, L. , Jiang‐xia, L. , & Guan‐qun, Y. (2017). The clinical value of early rescue intracytoplasmic sperm injection in conventional vitro fertilization and embryo transfer. Chinese Journal of Birth Health & Heredity, 25(06), 118–120. 10.13404/j.cnki.cjbhh.2017.06.049

[and14182-bib-0037] Pisarska, M. D. , Casson, P. R. , Cisneros, P. L. , Lamb, D. J. , & Carson, S. A. (1999). Fertilization after standard in vitro fertilization versus intractoplasmic sperm injection in subfertile males using sibling ooctes. Fertility & Sterility, 71(4), 627–632. 10.1016/S0015-0282(98)00538-X 10202870

[and14182-bib-0038] Saleh, A. , Kashir, J. , Thanassoulas, A. , Safiehgarabedian, B. , Lai, F. A. , & Nomikos, M. (2020). Essential role of sperm‐specific PLC‐Zeta in Egg activation and male factor infertility: an update. Frontiers in Cell and Developmental Biology, 8, 28. 10.3389/fcell.2020.00028 32064262 PMC7000359

[and14182-bib-0039] Sirard, M. , Richard, F. J. , Blondin, P. , & Robert, C. (2006). Contribution of the oocyte to embryo quality. Theriogenology, 65(1), 126–136. 10.1016/j.theriogenology.2005.09.020 16256189

[and14182-bib-0040] Topolcan, O. , Ulcova‐Gallova, Z. , Windrichova, J. , Losan, P. , & Topolcan, O. (2016). Anti‐Mullerian hormone in serum and seminal plasma in comparison with other male fertility parameters. Systems Biology in Reproductive Medicine, 62, 223–226. 27110929 10.3109/19396368.2016.1161864

[and14182-bib-0041] Van Calster, B. , & Vickers, A. J. (2015). Calibration of risk prediction models impact on decision‐analytic performance. Medical Decision Making, 35(2), 162–169. 10.1177/0272989x14547233 25155798

[and14182-bib-0042] Varghese, A. C. , Bragais, F. M. , Mukhopadhyay, D. , Kundu, S. , Pal, M. , Bhattacharyya, A. , & Agarwal, A. (2009). Human sperm DNA integrity in normal and abnormal semen samples and its correlation with sperm characteristics. Andrologia, 41(4), 207–215. 10.1111/j.1439-0272.2009.00917.x 19601931

[and14182-bib-0043] Verheyen, G. , Tournaye, H. , Staessen, C. , De Vos, A. , Vandervorst, M. , & Van Steirteghem, A. (1999). Controlled comparison of conventional in‐vitro fertilization and intracytoplasmic sperm injection in patients with asthenozoospermia. Human Reproduction, 14(9), 2313–2319. 10.1093/humrep/14.9.2313 10469701

[and14182-bib-0044] Vitek, W. , Galarraga, O. , Klatsky, P. C. , Robins, J. C. , Carson, S. A. , & Blazar, A. S. (2013). Management of the first in vitro fertilization cycle for unexplained infertility: A cost‐effectiveness analysis of split in vitro fertilization‐intracytoplasmic sperm injection. Fertility and Sterility, 100(5), 1381–1388. 10.1016/j.fertnstert.2013.06.035 23876534 PMC4503359

[and14182-bib-0045] Wang, C. , & Swerdloff, R. S. (2014). Limitations of semen analysis as a test of male fertility and anticipated needs from newer tests. Fertility & Sterility, 102(6), 1502–1507. 10.1016/j.fertnstert.2014.10.021 25458617 PMC4254491

[and14182-bib-0046] Yanagida, K. , Morozumi, K. , Katayose, H. , Hayashi, S. , & Sato, A. (2006). Successful pregnancy after ICSI with strontium oocyte activation in low rates of fertilization. Reproductive Biomedicine Online, 13(6), 801–806. 10.1016/s1472-6483(10)61027-9 17169199

[and14182-bib-0047] Yeste, M. , Jones, C. , Amdani, S. N. , Patel, S. , & Coward, K. (2016). Oocyte activation deficiency: A role for an oocyte contribution? Human Reproduction Update, 22(1), 23–47. 10.1093/humupd/dmv040 26346057

